# Microbial yield from physiotherapy assisted sputum production in respiratory outpatients

**DOI:** 10.1186/s12890-016-0188-2

**Published:** 2016-02-02

**Authors:** Philip J. Langridge, Reyenna L. Sheehan, David W. Denning

**Affiliations:** The National Aspergillosis Centre, ERC, 2nd floor, University Hospital South Manchester, Southmoor Road, Manchester, M23 9LT UK; The University of Manchester; Manchester Academic Health Science Centre, Manchester, UK

**Keywords:** Sputum, Induced, Physiotherapy, Aspergillus, Hypertonic

## Abstract

**Background:**

Sputum is a key diagnostic sample for those with chronic chest conditions including chronic and allergic aspergillus-related disease, but often not obtained in clinic.

The objective of this study was to evaluate physiotherapeutic interventions to obtain sputum from those not able to spontaneously produce and the subsequent microbiological result.

**Methods:**

Sputum samples were collected by physiotherapists from patients attending routine outpatient clinics managing their aspergillus-related diseases who were unable to spontaneously produce. Active Cycle of Breathing Techniques (ACBT) technique was applied first, for 10 min, followed by hypertonic saline induction using a Pari LC plus or Pari Sprint nebuliser, if necessary and deemed safe to do so. Samples processed in the laboratory using standard microbiological techniques for bacterial and fungal culture with the addition of *Aspergillus* real-time PCR.

**Results:**

Samples were procured from 353 of 364 (97 %) patients, 231 (65 %) by ACBT and 119 (34 %) with administration of hypertonic saline. Three of 125 (2.4 %) patients had significant bronchospasm during sputum induction. Sixteen patients’ sputum tested positive for *Aspergillus* culture, contrasting with 82 whose *Aspergillus* PCR was positive, 59 with a strong signal. PCR improved detection of *Aspergillus* by 350 %. Sputum from 124 (34 %) patients cultured other potentially pathogenic organisms which justified specific therapy.

**Conclusions:**

Physiotherapeutic interventions safely and effectively procured sputum from patients unable to spontaneously produce. The method for sputum induction was well-tolerated and time-efficient, with important microbiological results.

## Background

Induced sputum using nebulised saline to induce a productive cough has been studied for diagnosing *Pneumocystis* pneumonia (PCP) and pulmonary aspergillosis [1, 2]. Many patients attending clinics report they are not able to produce sputum spontaneously on request, having discarded their morning sputum. Yet a respiratory sample is critical for microbiological diagnosis of bacterial and fungal infections. Furthermore the yield of *Aspergillus* spp. from fungal cultures of sputum is poor and molecular diagnosis more sensitive, [3, 4], although improved means of processing specimens has been shown to improve culture yield [4, 5]. In patients with complex respiratory problems, multiple pathogens are common, the most common of which are *Streptococcus pneumoniae*, *Haemophilus influenzae*, *Pseudomonas aeruginosa* and *Aspergillus fumigatus*. Therapy of these different infections varies substantially and may be further influenced by resistance profiles. Hence accessing respiratory samples becomes an important part of clinical assessment and consequent improved outcomes, rather than relying on empirical choices, which are often unsuccessful.

The techniques for assisting sputum production include the Active Cycle of Breathing Techniques (ACBT) and sputum induction, prior to more invasive and costly bronchoscopy. Induced sputum production with nebulised hypertonic saline was reported to carry a 14-27 % rate of significant bronchospasm, [6, 7].

Many of the patients attending the National Aspergillosis Centre have complex respiratory problems with an average of 2.5 underlying respiratory conditions [8]. The microbiological yield, adverse events and general challenges of regular use of sputum production using ACBT and nebulised hypertonic saline in the outpatient setting has not been studied previously. This became possible in our service because of the routine contribution made by experienced physiotherapists in our aspergillosis clinics, employed expressly to contribute to infection diagnosis, as well as providing patient advice and training and administering/ assessing safety of nebulised antibiotics and antifungals.

Here we review our experience of physiotherapist-directed efforts to acquire sputum samples, the sputum production rates, adverse events and the microbiological yield. Our service has an extremely low rate of pulmonary tuberculosis (PTB), so we infrequently requested mycobacterial culture, despite our clinical observation that the relative rate of non-tuberculous mycobacterial (NTM) infection in chronic pulmonary aspergillosis (CPA) is higher than in general respiratory practice. We therefore cannot comment on the performance of these techniques on mycobacterial smear or culture yield. The focus is on rapidly growing bacteria, fungal culture and *Aspergillus* PCR.

## Methods

### Patients and clinics

Three hundred and sixty four patients aged 22-90 years on treatment for, or thought to have *Aspergillus* disease, including chronic pulmonary aspergillosis (CPA), allergic bronchopulmonary aspergillosis (ABPA), severe asthma with fungal sensitization (SAFS) and/or *Aspergillus* bronchitis (Table 1) were referred for sputum induction. All were attending the National Aspergillosis Centre in Manchester and were unable to spontaneously produce a sputum sample. These samples were sent for microbiological testing as directed by the physician. This report is a retrospective service evaluation of all patients who underwent physiotherapy-assisted sputum production in the outpatient clinics between 25/04/2012 and 23/04/2014 to assess sample yield and safety, and as such is exempt from ethical review. These physiotherapeutic interventions were performed as part of their standard care in clinic and consent for each intervention was obtained accordingly.

**Table 1:** Working clinical diagnoses in 364 patients

Diagnosis	No of patients with provisional or confirmed diagnosis
Chronic pulmonary aspergillosis	183
ABPA	58
ABPA and CPA	9
*Aspergillus* bronchitis	41
Single aspergilloma	5
Severe Asthma with Fungal Sensitisation	8
Asthma with fungal sensitisation	3
Subacute invasive aspergillosis	7
*Aspergillus* airway colonisation	1
*Aspergillus* pericarditis	1
*Aspergillus* sinusitis	1
*Candida* bronchitis	2
Other	45

*ABPA* allergic bronchopulmonary aspergillosis, *CPA* chronic pulmonary aspergillosis

### Disease definitions

The diagnosis of CPA was based primarily on antibody and radiological data, [8, 9], ABPA primarily on clinical and serological data, [10], SAFS as described previously, [11, 12] and *Aspergillus* bronchitis as recently revisited [13].

### Sputum production techniques

**Fig. 1:**
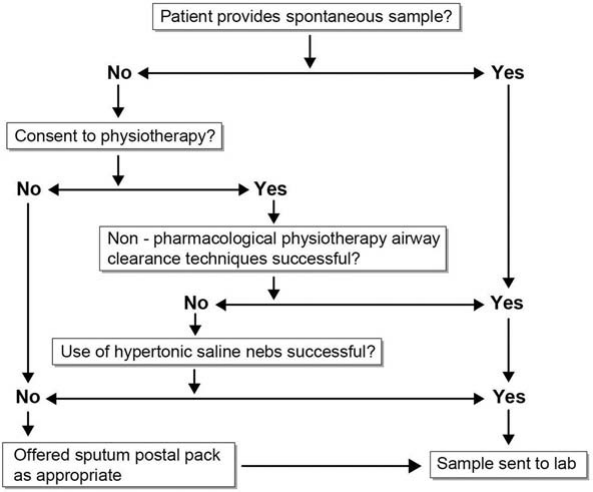
Method of procuring sputum samples

**Fig. 2:**
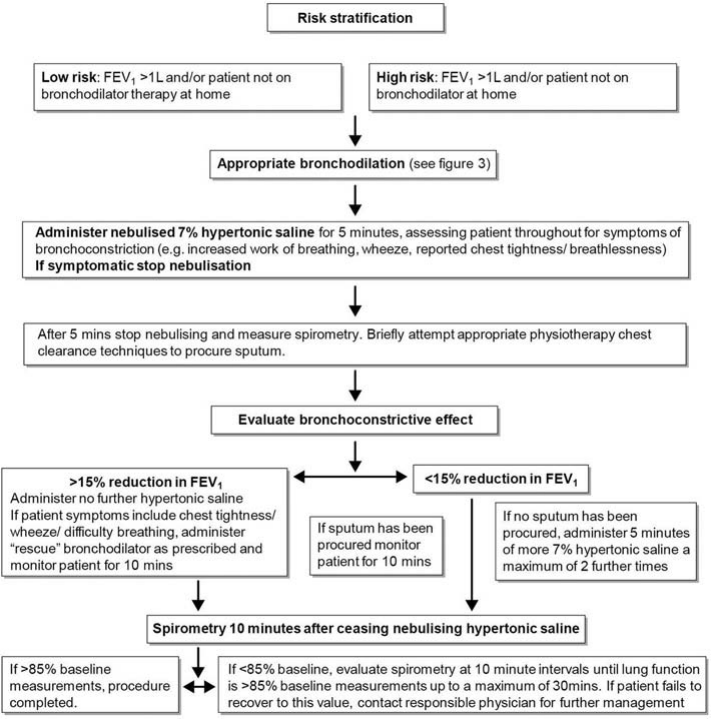
Method of sputum induction using hypertonic saline

**Fig. 3:**
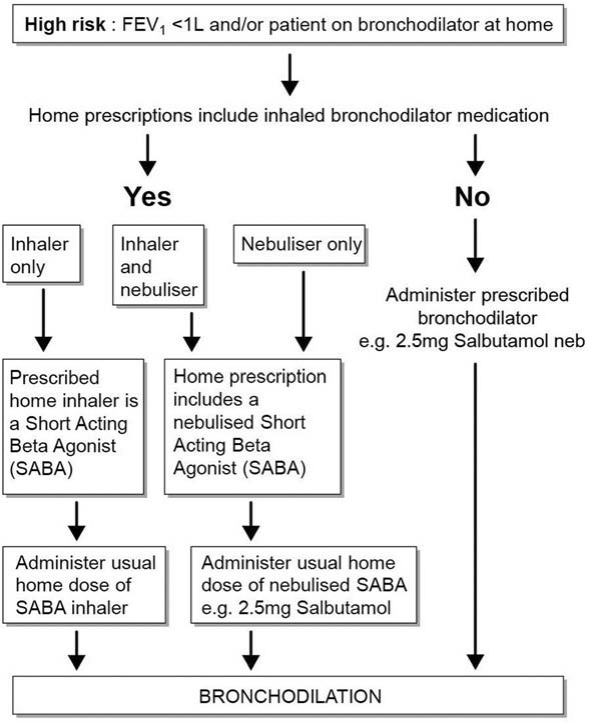
Bronchodilation pathway

After gaining consent, patients were firstly instructed in ACBT which was performed for 10 min (see Fig 1). If this was unsuccessful, consideration was given to nebulised hypertonic saline (7 % NaCl) to induce sputum (Figs. 1, 2 and 3). Previous intolerance of nebulised hypertonic saline, lack of consent, and/or perceived exceptionally high clinical risk (e.g. FEV1 < 0.5 L) excluded patients from induction with hypertonic saline. Hypertonic saline was administered via the breath enhanced Pari LC plus or Pari Sprint nebulisers driven by Clement Clarke’s Econoneb compressor. The patients excluded from sputum induction and unable to produce after 10 min of ACBT were offered alternative physiotherapeutic modalities including postural drainage, autogenic drainage and “bubble” positive expiratory pressure.

### Microbiological methods

Generally 2 samples were provided, one for microscopy with gram stain and bacterial and fungal culture, the other for DNA extraction and *Aspergillus*-specific PCR. Sputum was digested with Sputasol® (ratio 1:1), vortexed, a slide prepared for gram stain and 10 μL-streaked on two Sabouraud dextrose agar plates [14] and incubated at 30 °C and 37 °C for 7 days. DNA extraction was performed from 0.5–3 mL of sample using the MycXtra kit (Myconostica, Cambridge, UK). DNA was eluted in 40 μL of buffer S5 and 10 μL was used for quantitative PCR (qPCR) with the MycAssay Aspergillus kit (Myconostica) [3]. As per the manufacturer instructions, a Ct of >38 is negative, a Ct from 36-38 is a weak positive and <36 is interpreted as a strong positive. Susceptibility testing of *Aspergillus* isolates was routinely done and reported, as previously described [15].

## Results

Table 1 shows the working diagnoses of the patients on referral. Sputum was procured in 353 out of 364 patients (97 %) by ACBT (231 (65 %)) or hypertonic sputum induction 119 (34 %). Three of 125 (2.4 %) patients had significant bronchospasm during sputum induction. ACBT was unsuccessful in a further 8 patients who declined hypertonic sputum induction and sputum was not produced by 3 patients who underwent hypertonic sputum induction. Seven patients had sputa obtained from physiotherapists at multiple clinic dates. One patient, in the process of nebulised acetylcysteine challenge testing, produced sputum. Another patient required aseptic endotracheal suction via tracheostomy to gather sputum. ACBT took about 15 min per patient and if ACBT was followed by hypertonic saline induction, which took ~25 min per patient.

Several organisms were cultured from sputum samples (Table 2). One hundred and twenty three samples were culture positive - 56 probably significant bacteria, including one *Mycobacteria avium intracellulare,* 16 *Aspergillus* spp and 51 *Candida* spp., *Saccharomyces cerevisiae* or other probably insignificant yeasts. Among the bacteria were two patients with MRSA, 19 with *Pseudomonas aeruginosa* and 2 with *Stenotrophomonas maltophilia*, classically organisms that do not respond to standard antibiotics for community acquired pneumonia.

**Table 2:** Microbiological (culture) yield by organism

	Non-pharmacological physiotherapy airway clearance techniques	Hypertonic saline
Probably significant organisms		
*Aspergillus fumigatus* complex	12	3
*Aspergillus terreus*	1	0
*Haemophilus influenzae*	9	5
*Haemophilus parainfluenzae*	1	0
*Staphylococcus aureus*	2	2
Methicillin resistant *Staphylococcus aureus*	2	0
*Escherichia coli*	1	1
*Pseudomonas aeruginosa*	16	3
*Pseudomonas eurefenosa*	1	0
*Mycobacterium Intracellulare*	0	1
*Moraxella catarrhalis*	1	1
*Enterobacter cloacae*	0	1
*Streptococcus pneumoniae*	3	0
*Citrobacter koseri*	1	1
*Serratia marcescens*	1	0
*Stenotrophomononas maltophilia*	2	0
*Acinetobacter haemolyticus*	1	0
Probably insignificant organisms		
*Saccharomyces cerevisiae*	2	0
*Candida albicans*	16	7
*Candida glabrata*	7	3
*Candida lusitaniae*	0	1
*Candida tropicalis*	1	1
*Candida parapsilosis*	1	0
*Unidentified fungus*	5	1
*Yeasts unspecified*	4	2
Total	91	33

Of the 3 methods used to detect *Aspergillus*, only 18 patients’ sputum showed fungal elements on microscopy consistent with *Aspergillus* spp. and 16 grew *Aspergillus* in culture (Tables 2 and 3). Culture was slightly more often positive from ACBT samples (5 %) than hypertonic induced sputum (2 %), but this was not significant by Fisher Exact test (*p* = 0.28). Of the 18 microscopy positive samples, only 7 of these tested positive for *Aspergillus* PCR, consistent with other fungi being implicated in symptoms (one was *Scedosporium apiospermum*), *Candida* pseudohyphae being seen or contaminated microscopy materials. Eighty-two samples were *Aspergillus* PCR positive (Table 3), of which 59 (72 %) had strong signals; four had a technical failure. Twenty three samples were positive by PCR and culture and/or microscopy. Of the 74 samples negative for both fungal culture and microscopy, fungal PCR was strongly positive in 37 (50 %). There was a slightly higher frequency of strong signals from sputum obtained by ACBT than with induced sputum (76 % versus 64 %), but this was not significant by chi-square (p = 0.27).

**Table 3:** Success in yielding sputum from 364 patients with the 2 techniques used and the microbiological results obtained

	Physiotherapy techniques (%)	Induced sputum (%)	Totals (%)
Total number patients treated	239	125*	364
Unable to procure sputum from patient	8 (3)^a^	3 (2)^a^	353 (97
Sputum induction discontinued due to adverse effects	0	3 (2)^b^	3 (1)
Positive bacterial and/or fungal culture	76 (32)	29 (23)	105 (30)
Positive *Aspergillus* PCR	54 (23 %)	28 (22 %)	82 (23)
Strongly positive *Aspergillus* PCR (Ct <36)	41 (76 %)	18 (64 %)	58 (16)
Aspergillus cultured	13 (5 %)	3 (2 %)	16 (5)
Hyphae consistent with *Aspergillus* spp. seen on sputum microscopy	11 (5 %)	7 (6 %)	18 (5)

* 1 patient required N-acetylcysteine. ^a^ patient declined; ^b^ patients wheezy

*PCR* polymerase chain reaction

Of the 16 *Aspergillus* isolates grown, 3 were not referred for susceptibility testing. The *A. terreus* isolate was resistant to amphotericin B (minimum inhibitory concentration (MIC) = >8 mg/L), and susceptble to itraconazole, voriconazole and posaconazole. Nine isolates were fully susceptible. One *A. fumigatus* strain was solely resistant to voriconazole (MIC >8 mg/L) and two were panazole resistant, with MICs to all 3 azoles of >8 mg/L, and susceptible to amphotericin B. Overall, therefore 4 (31 %) isolates were resistant to one or more drugs.

## Discussion

The interventions from the specialist physiotherapists were well tolerated and successful in procuring sputum for testing. On site sampling led to timely processing. Eight patients failed to produce sputum using physiotherapeutic techniques but were unsuitable to go on to have sputum induction using hypertonic saline (e.g. time constraints, high clinical risk, lack of consent). Our fall-back position for these patients is to given them a sterile pot for expectoration, with special packaging, pre-paid addressed plastic envelope and request form (“postal pack for sputum”), which is also successful, although slower.

Little has been published about induced sputum and resultant bacterial culture. One study of 48 children with CF showed that in 2 cases samples induced with nebulised 7 % saline grew additional organisms compared with the prior spontaneous sample [16]. Positive bacterial culture may alter empirical antibiotic treatment or prompt further investigation. For example, gram negative cultures may prompt intravenous antibiotic treatment or further investigation for bronchiectasis diagnosis.

Empirical antifungal therapy for *Aspergillus* is rarely given in the outpatient setting. Arguably finding *Aspergillus* (by whatever method) has a potentially profound impact on management. While colonization of the airway is more common in certain settings such as COPD or cystic fibrosis, finding *Aspergillus* usually means some form of aspergillosis [17, 18]. Here culture was positive in 16 patients, whereas PCR was positive 72 patients, with a strong signal in 59, an improved yield of 360 %. Monitoring sputum fungal loads with the strength of PCR signal aids treatment efficacy monitoring [19]. Unlike with PCP, [20] the cost-effectiveness of sputum induction for *Aspergillus*-related disease has not been estimated. It is expected that sputum induction in the management of *Aspergillus*-related disease is likely to be cost-effective when compared to bronchoalveolar lavage, and may have a higher yield [4].

### Adverse effects

Non-pharmacological physiotherapy airway clearance techniques were extremely well tolerated with no reported problems. Two hundred and thirty nine patients needed only this intervention to produce sputum, which required short treatment times of up to 10 min.

One hundred and twenty five patients underwent hypertonic saline challenge for sputum induction. Three (2 %) of those induced with hypertonic saline experienced bronchospasm, all of whom required rescue with nebulised bronchodilator. All patients returned home safely the same day of their clinic appointment. In a previous service evaluation, of those adult non-CF patients challenged with 4 ml 7 % saline, 17 % of subjects showed an initial >15 % drop in FEV_1_ reaction to inhalation of 4 ml 7 % saline. Makris *et al.* [6] report an incidence of bronchospasm of approximately 27 % when performing sputum induction with COPD patients. They found 31 % presented with a hyperresponsive (>20 % drop in FEV_1_) reaction to inhalation of 4.5 % saline, despite a preceding dose of 200 μg salbutamol via metered dose inhaler. This may be partly accounted for by the fact that in their study there was a 4 week washout period with no inhaled/oral steroid use, and long acting bronchodilators and short acting bronchodilators were omitted 12 and 8 h respectively before interventions. In our service evaluation, patients had taken their usual steroid/ bronchodilator medication which accounts for the low rate of associated bronchospasm. Also, in our service evaluation the patients received 5 min inhalations of 7 % saline, approximating to a dose of 2 ml over the 5 min. This lower dose, although sufficient to yield sputum for sampling, may not have been enough to precipitate bronchospasm. Some patients, however, received 5 min of 7 % saline inhalations repeated 3 times (total approximate dose 6 ml) with no duly associated increased adverse effects.

### Clinical impact of sputum testing

Patients with MRSA (*n* = 2), *Pseudomonas aeruginosa* (*n* = 19) and *Stenotrophomonas maltophilia* (*n* = 2) require alternative antibiotics and often dose escalation for successful eradication. These are therefore important findings. The diagnosis of *Aspergillus* bronchitis requires repeated identification of *Aspergillus* species in the airway, by whatever means (13). Forty one patients studied had this diagnosis. A stronger PCR signal implies disease rather than colonisation [17]. In the context of patients taking oral antifungal azole therapy with therapeutic antifungal levels, a strong PCR signal probably signifies triazole antifungal resistance.

Tolerance of nebulised hypertonic saline in the context of sputum induction may provide helpful reassurance about long term use for patient’s needing this intervention of chronic disease management (e.g. bronchiectasis and ABPA) [21].

### Limitations

The patients had mixed respiratory diagnoses, reflecting real life clinic experience. It was impractical to retrospectively evaluate the clinical impact of 364 physiotherapy interventions without additional resources. It was not noted *a priori* what samples were obtained per patient at time of collection: the results were examined retrospectively from an electronic pathology reporting system (SunQuest ICE). For the first year of the study date, the sputum requesting was done using paper forms so there is no accessible audit trail to differentiate what was sent for processing and what results actually were: there may have been lost and/or insufficient samples. Very few samples were submitted for mycobacterial culture and acid fast bacillus microscopy.

The ACBT, when used, was tailored specifically to the clinical presentation at the time by the physiotherapist so that the interventions may differ subtly in terms of techniques used, repetitions etc. Also, the dose of hypertonic saline administered did vary between patients according to when sputum was produced.

Choice of nebuliser/compressor may influence delivery of hypertonic saline [22]. However, a recommendation that ultrasonic nebulisers should be used due to usually inadequate outputs from other nebulisers [23, 24] no longer necessarily applies [25, 26]: the output from the Pari Sprint nebuliser is 590 mg/min, mass median diameter 2.9 μg and 75 % particles below 5 μm (when driven by PARI Boy® SX compressor). Practicalities inherent to ultrasonic nebulisers (e.g. access, cleaning, cost) may steer clinicians to other equipment, especially when doing so still results in the desired outcome *viz.* sputum sample procurement.

The equipment used was what was readily accessible in the clinic. When routinely tested by the medical engineering department, the compressor flow rates varied between 7.5 and 9.5 Lpm despite being the same makes/ models. This variance in flow rate would result in varying nebuliser outputs [27].

The goal of the interventions was to elicit sputum from those who could not spontaneously produce. It is not known whether sputum gained from ACBT yielded more clinically-relevant information than that elicited after inhalation of 7 % hypertonic saline. Neither is it known whether unsuccessful airway clearance techniques reduced the time or dose required for subsequent nebulised hypertonic saline to produce a sputum sample. Elkins *et al* [20] evaluated the effect of airway clearance techniques as part of sputum induction: they showed organism identification did not improve with them, but the difference in sensitivities of the tests was 7 % better with airway clearance techniques. It is also not known from this evaluation if there is an order effect on success of testing: if 2 sputum samples were produced the first one could have been sent for culture, the second for PCR or vice-versa. It is also not known how spontaneous samples tested compared to physiotherapist-collected samples. It is recommended that future work investigates the order effect of sputum sampling and that physiotherapist-collected samples are compared to spontaneously-produced ones when subjected to fungal testing.

## Conclusion

Physiotherapeutic interventions safely and effectively procured sputum from patients unable to spontaneously produce. The method for sputum induction was well-tolerated and time-efficient, with important microbiological results. Molecular detection of *Aspergillus* spp. was superior to culture, although resistance was found in 31 % of those that were cultured. Sputum from 34 % patients cultured other potentially pathogenic organisms which justified specific therapy.

## Abbreviations

ABPA: allergic bronchopulmonary aspergillosis
ACBT: active cycle of breathing technique
CF: cystic fibrosis
CPA: chronic pulmonary aspergillosis
Lpm: Litres per minute
MIC: minimum inhibitory concentration
MRSA: methicillin resistant *Staphylococcus aureus*
NTM: non-tuberculous mycobacterial infection
PCP: *Pneumocystis* pneumonia
PCR: polymerase chain reaction
SAFS: severe asthma with fungal sensitization
